# Surviving and thriving after breast cancer treatment

**DOI:** 10.5694/mja2.51671

**Published:** 2022-08-14

**Authors:** Christobel M Saunders, Lesley Stafford, Martha Hickey

**Affiliations:** ^1^ University of Melbourne Melbourne VIC; ^2^ Royal Melbourne Hospital Melbourne VIC; ^3^ Royal Women's Hospital Melbourne VIC

**Keywords:** Breast neoplasms, Quality of life

Breast cancer commonly causes significant physical and psychological sequelae; guidelines and models of care exist to ameliorate these

Breast cancer is the most diagnosed cancer in humans.^1^ In 2020, there were 2.3 million women diagnosed with breast cancer, and 685 000 deaths globally. In Australia, over 20 000 people were diagnosed in 2021.^2^

Currently, nearly 8 million people^3^ are alive who were diagnosed in the past 5 years, making breast cancer the world’s most prevalent cancer. Moreover, there are more disability‐adjusted life years lost to breast cancer globally than any other type of cancer.^3^ Thus, any morbidity in a group of people this size is likely to have profound impacts at a societal as well as an individual level.

In breast cancer, like most cancers, prevention remains elusive. We therefore focus on reducing its impact by early detection through awareness and screening, and optimising treatments that improve survival — over 90% of Australians diagnosed with breast cancer will survive.^2^ However, survivors may experience reduced quality of life. Increased emphasis on improving quality of life has led to re‐evaluating use of toxic treatments which have minimal survival impacts and developing more targeted treatments. Targeted treatments include surgical techniques such as oncoplastic surgery and less invasive axillary surgery, targeted and hypofractionated radiotherapy, and targeted drug treatments. In addition, cancer care delivery has improved with access to specialist nurses, counsellors, peer support and allied health, often driven by strong patient advocacy.^4^ Despite this, we know that the physical and psychological consequences of breast cancer can be profound and there remain significant impacts of the diagnosis and treatment on physical, emotional and functional wellbeing.^5^ In parallel is increasing complexity of care, involving many disciplines and therapies, with many opportunities for the patient to become lost in the system, leading to inefficient care.^6^, ^7^

Despite a large body of evidence outlining the challenges breast cancer survivors face, many continue to have unmet needs.^8^ This may simply be because we do not systematically measure and respond to many of the problems faced by patients after their diagnosis. The potential of patient‐reported outcome measures to improve the care of cancer survivors is increasingly recognised because they allow accurate measurement of a range of outcomes through the patient’s lens and throughout a patient’s clinical journey. The collection of patient‐reported outcome measures has been encouraged in the 2020–25 National Health Reform Agreement to empower patient involvement in their care, improve care across the health system, and enable clinicians to focus on outcomes that matter most to patients.^9^

If we recognise that breast cancer survivors face many challenges and would benefit from ongoing interventions that address their survivorship needs, we need effective methods to measure these challenges and needs, evidence‐based management approaches, and resources to find better care options.

## Psychosocial issues after breast cancer

The literature consistently shows that anxiety is significantly higher among breast cancer survivors than other women in the community, and depression and other mood disturbances are more common in survivors and their spouses.^10^ Patients with previous psychological or psychiatric morbidity are more likely to suffer from recurrent psychological distress, indicating a potential target group for preventive interventions.^11^

Accurately assessing symptoms and addressing them with proven interventions^12^ may include formal psychological referral (often under‐resourced) or for less significant distress, practices such as yoga, meditation and mindfulness.^13^, ^14^ Guidelines for both specialists and general practitioners are making inroads into better after‐care for those who have had cancer.^15^

## Fear of cancer recurrence

Fear of recurrence is common and often long lasting, manifesting as constant and intrusive thoughts about cancer, regardless of actual prognosis or risk of recurrence, and can cause increased use of health services and non‐adherence to treatment and follow‐up.^16^ Side effects of treatment, a constant reminder of cancer, can exacerbate fear of recurrence. Discussing fear of recurrence with patients and accessing help if needed can significantly improve quality of life and ameliorate morbid fears. Evidence‐based national guidelines have been developed.^16^

## Fatigue

Around 30% of women experience fatigue after breast cancer, especially those who have received chemotherapy.^5^ This can be prolonged and is thought to be driven by both physical and psychological factors, and may affect mental health.^5^ Exercise has the strongest evidence base for the management of fatigue in patients with cancer, and referral to an exercise physiologist or physiotherapist to prescribe a personalised regimen is often warranted.^18^ Insomnia is a lesser recognised but often debilitating issue for many breast cancer survivors, and interventions such as cognitive behaviour therapy show promise.^19^

## Vasomotor symptoms

The extended use of endocrine therapies such as aromatase inhibitors for up to 10 years, as well as the gonadotoxic effects of chemotherapy, mean a very high proportion of women who have had breast cancer will suffer menopausal symptoms, including hot flushes and night sweats, poor concentration and sleep, vaginal dryness and emotional changes.^20^ Managing these with menopausal hormone therapy is usually contraindicated.^20^ Topical vaginal oestriol is useful and probably safe, particularly in women who are not receiving aromatase inhibitors.^21^

Australian guidelines outline a wide range of pharmacological and non‐pharmacological interventions and the strength of evidence for their safety and efficacy.^21^ Other important resources are the dedicated public menopause clinics for cancer patients which exist in a number of major cities including Perth and Melbourne (https://www.kemh.health.wa.gov.au/For‐Health‐Professionals/Gynaecology/Menopause; https://www.thewomens.org.au/patients‐visitors/clinics‐and‐services/menopause/menopause‐symptoms‐after‐cancer/).

## Sexual issues

Sexual difficulties after breast cancer are extremely common. These are experienced by over 70% of women,^22^ and range from those caused by alteration in body image and physical pain or movement restriction, to endocrine side effects including vaginal dryness, dyspareunia and loss of libido. While simple treatments such as vaginal moisturisers may be helpful^23^ for some, many will require more involved treatment.^23^ However, access to sexual counsellors and menopause services is limited to very few public or private services and is mainly available in metropolitan areas in Australia.

## Cognitive impairment

“Chemo brain” is one of the commonest and most debilitating symptoms reported by women not only during and after chemotherapy but also while receiving endocrine therapies.^24^, ^25^ The effects on attention, learning and memory can affect social and work performance. To date, no specific treatments have proven efficacy, but the National Comprehensive Cancer Network survivorship guidelines suggest that addressing related factors such as menopause symptoms or depression can be helpful.^26^

## Financial impacts

Increasing recognition is being placed on the financial impacts of cancer — both medical and non‐medical out‐of‐pocket expenses. Over a quarter of women will spend more than $17 000 on treatment, and for some this will prove catastrophic.^27^ Moreover, non‐medical out‐of‐pocket expenses such as lost time at work, childcare and travel for treatment are equally as important. Informed financial consent and reducing the impact of costs are major advocacy issues for both patient organisations and governments, and further development of the Australian Government medical costs finder website (https://www.health.gov.au/resources/apps‐and‐tools/medical‐costs‐finder) may help address some of the informational needs.

## Other physical symptoms

Lymphoedema, chronic pain, bone loss, cardiovascular toxicity (from both chemotherapy and radiotherapy), body image concerns, particularly if access to reconstruction has been challenging, and loss of fertility are some of the many other impacts of breast cancer.^12^ The needs of younger and older patients may be very different.

## Moving forward

Current inadequacies in service provision and out‐of‐pocket costs can make support hard for patients to access, although chronic care plans seek to address this somewhat. Newer models of care such as nurse‐led clinics, care navigation, telehealth, and online support and survivorship plans may be able to bridge this gap.^28^ However, we always need to be mindful that these models must meet the needs of all patients not just those with already good health literacy, and the place of care plans across the cancer journey is still not clear.^29^ A holistic but tailored approach to care and survivorship, including careful assessment of issues and developing survivorship care plans to help tackle them,^28^ is needed. The use of cancer care navigators, breast care nurse consultants who lead follow‐up, and shared care with general practitioners are important steps that we should be aiming for in practice to maximise a patient’s ability to thrive after cancer. Multidisciplinary care during the active treatment phase of breast cancer is the norm — it now needs to extend to survivorship.

## Conclusion

Every experience of breast cancer will vary, but for the majority there will be some long‐lasting disease and treatment side effects which will impair a return to full health and diminish physical, social, psychological, sexual and occupational wellbeing. If a patient who is unable to tolerate treatment ceases it early, this can potentially affect survival. The key is communicating with the patient and treatment team, including the GP, regarding potential side effects, early identification, and effective interventions.

## Competing interests

Christobel Saunders receives speaker fees from Merck Sharp and Dohme.

## Provenance

Commissioned; externally peer reviewed.

## Open access

Open access publishing facilitated by The University of Melbourne, as part of the Wiley – The University of Melbourne agreement via the Council of Australian University Librarians.
